# Ultrasound-based deep learning radiomics for enhanced axillary lymph node metastasis assessment: a multicenter study

**DOI:** 10.1093/oncolo/oyaf090

**Published:** 2025-05-11

**Authors:** Di Zhang, Wang Zhou, Wen-Wu Lu, Xia-Chuan Qin, Xian-Ya Zhang, Yan-Hong Luo, Jun Wu, Jun-Li Wang, Jun-Jie Zhao, Chao-Xue Zhang

**Affiliations:** Department of Ultrasound, The First Affiliated Hospital of Anhui Medical University, Hefei, China; Department of Ultrasound, The First Affiliated Hospital of Anhui Medical University, Hefei, China; Department of Ultrasound, The First Affiliated Hospital of Anhui Medical University, Hefei, China; Department of Medical Ultrasound, Chengdu Second People’s Hospital, Chengdu, China; Department of Medical Ultrasound, Tongji Hospital, Tongji Medical College, Huazhong University of Science and Technology, Wuhan, China; Department of Ultrasound, The Third Affiliated Hospital of Anhui Medical University, Hefei First People’s Hospital, Hefei, China; Department of Ultrasound, The Second Affiliated Hospital of Anhui Medical University, Hefei, China; Department of Ultrasound, WuHu Hospital, East China Normal University (The Second People’s Hospital, WuHu), Wuhu, China; Department of Medical Ultrasound, Fuyang Cancer Hospital, Fuyang, China; Department of Ultrasound, The First Affiliated Hospital of Anhui Medical University, Hefei, China

**Keywords:** breast cancer, axillary lymph node metastasis, radiomics, ultrasound, predictive model

## Abstract

**Background:**

Accurate preoperative assessment of axillary lymph node metastasis (ALNM) in breast cancer is crucial for guiding treatment decisions. This study aimed to develop a deep-learning radiomics model for assessing ALNM and to evaluate its impact on radiologists’ diagnostic accuracy.

**Methods:**

This multicenter study included 866 breast cancer patients from 6 hospitals. The data were categorized into training, internal test, external test, and prospective test sets. Deep learning and handcrafted radiomics features were extracted from ultrasound images of primary tumors and lymph nodes. The tumor score and LN score were calculated following feature selection, and a clinical-radiomics model was constructed based on these scores along with clinical-ultrasonic risk factors. The model’s performance was validated across the 3 test sets. Additionally, the diagnostic performance of radiologists, with and without model assistance, was evaluated.

**Results:**

The clinical-radiomics model demonstrated robust discrimination with AUCs of 0.94, 0.92, 0.91, and 0.95 in the training, internal test, external test, and prospective test sets, respectively. It surpassed the clinical model and single score in all sets (*P* < .05). Decision curve analysis and clinical impact curves validated the clinical utility of the clinical-radiomics model. Moreover, the model significantly improved radiologists’ diagnostic accuracy, with AUCs increasing from 0.71 to 0.82 for the junior radiologist and from 0.75 to 0.85 for the senior radiologist.

**Conclusions:**

The clinical-radiomics model effectively predicts ALNM in breast cancer patients using noninvasive ultrasound features. Additionally, it enhances radiologists’ diagnostic accuracy, potentially optimizing resource allocation in breast cancer management.

**Implications for Practice**

This study introduces a novel deep-learning radiomics model that enhances the preoperative assessment of axillary lymph node metastasis (ALNM) in breast cancer by integrating tumor and lymph node imaging data. The model significantly improves diagnostic accuracy compared to traditional methods and can further assist radiologists in their evaluations. Its application could lead to more informed treatment decisions, reduce unnecessary procedures, and optimize resource allocation in clinical settings.

## Introduction

Breast cancer is the most common malignant tumor among women worldwide and a leading cause of cancer-related deaths.^1^ The status of the axillary lymph node (ALN) is a critical factor in determining prognosis, staging breast cancer, and selecting the most appropriate treatment plan.^2-4^ In clinical practice, sentinel lymph node biopsy (SLNB) and ALN dissection (ALND) are the standard methods for assessing ALN status. However, these invasive procedures carry the risk of complications, such as lymphedema, infection, limited shoulder mobility, and damage to major blood vessels and nerves.^5,6^ Furthermore, 43%-65% of patients with positive sentinel lymph nodes (LNs) undergo unnecessary axillary surgery due to the absence of additional non-sentinel lymph node metastasis, resulting in overtreatment and high morbidity rates.^7^ Therefore, developing an accurate, noninvasive preoperative method for assessing ALN status is essential for individualized clinical treatment strategies and reducing unnecessary lymph node dissection.

Ultrasound (US), due to its low cost and non-radiation, is widely used for the preoperative diagnosis of breast lesions and the assessment of ALN status.^8^ Previous studies have demonstrated that specific US characteristics of primary breast cancer, including tumor size, calcifications, and architectural distortion, are correlated with ALN metastasis (ALNM).^9,10^ Research has also confirmed that axillary US offers important insights into the status of ALN in breast cancer.^11^ However, the US primarily offers visual images and relies on qualitative analysis of tumors. Diagnostic performance in detecting ALN involvement is heavily dependent on the radiologist’s expertise, resulting in significant inter-observer variability.^12^

Fortunately, the emergence of radiomics has created new opportunities for image analysis. Radiomics transforms medical images into quantitative data by extracting high-throughput features, revealing tumor heterogeneity, and offering potential noninvasive biomarkers to aid clinical decision-making.^13-15^ Recent studies have demonstrated that deep learning radiomics, which combines deep learning features automatically learned by convolutional neural network with handcrafted radiomics features, has shown excellent performance in predicting ALN status in breast cancer.^16-19^ However, these studies have focused exclusively on radiomics features extracted from tumors. LNs, which are the true targets for predicting lymph node metastasis, have not yet been considered. Moreover, these studies did not evaluate the practical advantages of deep learning radiomics in prospective diagnostic settings or explore its potential to improve radiologists’ diagnostic accuracy.

Therefore, this study aims to establish and validate a deep-learning radiomics model based on US-derived radiomics features from both the primary tumor and ALN, enabling noninvasive preoperative prediction of ALNM in breast cancer patients. We also validate the applicability of the artificial intelligence (AI) model as a useful tool to assist radiologists in diagnosing ALNM and evaluate its impact on supporting radiologists’ decision-making.

## Materials and methods

This prospective-retrospective multicenter study employed a 4-phase validation framework: (1) model development using retrospective data, (2) internal dataset validation, (3) external multicenter validation, and (4) prospective clinical utility assessment.

### Research subjects

Between February 2012 and May 2024, individuals diagnosed with primary breast cancer through operative histopathological assessment at 6 collaborating hospitals were enrolled. The participating institutions included The First Affiliated Hospital of Anhui Medical University, Hefei First People’s Hospital, Fuyang Cancer Hospital, The Second Affiliated Hospital of Anhui Medical University, Nanchong Central Hospital and Wuhu Hospital Affiliated with East China Normal University. All participating centers are tertiary hospitals in China with accredited breast ultrasound departments. Institutional selection criteria are detailed in **Supplementary Material**. Patient inclusion criteria, as specified in **Supplementary Material**, with core selection requirements including (1) preoperative ultrasound within 2 weeks of surgery; (2) histologically confirmed primary IBC; (3) definitive ALN status through SLNB/ALND. The patient enrollment process is shown in **Figure S1**.

Finally, 866 eligible breast cancer patients from 6 hospitals were included for training and testing. Of these, 527 patients from Hospital 1 (The First Affiliated Hospital of Anhui Medical University) were enrolled. These patients were randomly split into a training set (421 patients) and an internal test set (106 patients) in an 8:2 ratio. According to the same standard, 222 patients from the remaining 5 hospitals were included in the pooled external test set. An additional 107 patients were prospectively tested at Hospital 1 between March 10, 2024, and May 2024. The detailed distribution of patient samples across the participating hospitals is provided in **Table S1**.

### Clinic-pathologic data and US image collection

The ALN status was assessed through either SLNB or ALND. The baseline clinicopathological data, sourced from patient medical records, included age, tumor size, histologic type, immunohistochemistry (IHC) results, and postoperative status of ALNs. Additionally, for the prospective test set, patients’ clinical symptoms were collected prior to the US examination for subsequent analysis by the radiologist. The breast US images were obtained from the imaging archives of the 6 institutions participating in this study.

Detailed information on the US examination procedures and feature evaluation can be found in **Supplementary Material**. Only the most recent preoperative breast US examination was analyzed for patients who had undergone multiple examinations. The status of ALNs reported in the US was derived from US reports. Axillary US images highlighting key characteristics of suspicious ALNs were archived in the Picture Archiving and Communication Systems for subsequent analysis and validation. These records were retrospectively evaluated and confirmed by 2 radiologists, each with over 18 years of experience in breast US. Metastatic ALNs were identified on US if at least one of the following criteria was met: (1) long-axis/short-axis diameter ratio <2; (2) cortical thickness >3.5 mm; (3) complete or partial loss of fatty hilum; (4) color doppler imaging shows non-hilar cortical blood flow; (5) LNs are completely or partially replaced by poorly circumscribed or asymmetrical masses, and microcalcifications are present within the LNs.^20,21^ Expert radiologists reviewed both the US reports and the images to confirm the ALN status observed on US.

### Lesion segmentation and feature extraction

Initially, a radiologist with 9 years of experience in breast imaging manually delineated the primary tumor and ipsilateral ALN using ITK-SNAP software (Version 3.8.0), unaware of the pathologic information. The region of interest (ROI) was manually defined on the image showing the largest section of the primary tumor and the most suspicious ALN for metastasis on the same side of the axilla. Handcrafted features were then automatically extracted using the Pyradiomics package in Python. For deep learning feature extraction, we implemented a deep convolutional neural network based on the VGG19 architecture. Detailed information on the feature extraction process can be found in **Supplementary Material**.

To guarantee the robustness and reproducibility of the extracted features, we assessed the consistency of the tumor and LN region delineations using the interclass correlation coefficient (ICC). Specifically, another radiologist with 6 years of experience, along with the original radiologist after a 2-week interval, re-segmented fifty randomly selected cases from the training set. The ICC was calculated to quantify the agreement between the 2 radiologists’ segmentations. Features with lower ICC values were considered to have poor consistency and were excluded from further analysis to maintain the reliability of the data. For clarity, the ICC values were categorized as follows: ICC ≥ 0.80 was considered excellent consistency; ICC between 0.60 and 0.79 was considered good consistency; ICC < 0.60 was considered poor consistency.

After evaluating the ICC values for all extracted features, those with an ICC lower than 0.80 were discarded from the feature set. This step ensured that only stable, reproducible features remained for further analysis.

### Radiomics score building

Radiomics scores were constructed for both the primary tumor and ALN ROIs. The process involved several key steps: (1) assessing feature reproducibility to evaluate inter-observer and intra-observer consistency, (2) retaining a comprehensive set of representative features based on mutual information, and (3) constructing the scores. Subsequently, separate radiomics scores were developed for the primary tumor (tumor score) and LN (LN score), both serving as predictors of ALN status. **Supplementary Material** provides detailed information on feature preprocessing and selection.

### Model construction

A univariate analysis was first performed to identify clinical-ultrasonic factors that significantly correlate with ALNM in the training set. Following this, we employed a stepwise backward multivariate regression approach, with a statistical significance threshold of *P* < .05, to identify independent predictors of ALNM among the 2 radiomics scores and clinical-ultrasonic risk factors. This process led to the construction of a clinical-radiomics model. To facilitate the visualization of this model, a nomogram was generated. For clinical application, a Nomo-score was derived for each patient by summing the points corresponding to the predictors in the clinical-radiomics model. Additionally, a clinical model incorporating only clinical-ultrasonic characteristics was developed using the same methodology for comparison.

### Radiologist assessment and AI-assisted assessment

To assess the potential benefit of our AI model in enhancing radiologists’ diagnostic accuracy, 2 radiologists (a junior radiologist with 5 years of experience and a senior radiologist with 15 years of experience) independently reviewed all US data (including primary tumors and LNs) and clinical information (age and clinical symptoms) of patients in the prospective test set, without knowledge of the pathology results. Prior to the AI-assisted assessment, both radiologists underwent a brief training session to familiarize themselves with the AI model’s predictions and interpretation. This training included an overview of the model’s output, case examples, and guidance on how to integrate the AI predictions into their diagnostic workflow.

The radiologists independently assessed the ALN status of the patients according to the aforementioned criteria by identifying the 5 US features of ALNs. Two weeks later, the same radiologists re-evaluated the ALN status with the assistance of the AI model’s predictions. They had the option to revise their initial diagnoses or retain them. The original assessments were then compared with the AI-assisted assessments in the prospective test set to determine whether the model improved the radiologists’ diagnostic accuracy.

### Statistical analysis

The statistical analyses were conducted utilizing R software version 4.2.2, MedCalc version 20.100, and SPSS software version 24.0. The calibration of the clinical-radiomics model was evaluated utilizing the Hosmer-Lemeshow test. To determine the clinical utility across different threshold odds, we employed the clinical impact curve. The predictive performance of various models and radiologists was assessed using the area under the receiver operating characteristic curve (AUC), with differences in AUC compared using the DeLong test. In addition, decision curve analysis (DCA) was used to demonstrate the net benefit of each model in clinical decision-making. The improvement in predictive accuracy for the clinical-radiomics model was measured using net reclassification improvement (NRI) and integrated discrimination improvement (IDI). Statistical significance was defined as 2-sided with *P* < .05. Details on the packages of R software can be found in **Table S2**.

## Results

### Clinicopathological characteristics

**Table 1** outlines the baseline information of patients and clinical-ultrasonic characteristics of breast lesions across the training, internal test, external test, and prospective test set. The mean ages of patients in the training, internal test, external test, and prospective test sets were 54.0 ± 10.6 years, 52.5 ± 8.8 years, 54.4 ± 11.6 years, and 53.8 ± 10.0 years, respectively. The proportions of ALNM cases were 64.6% (272/421), 67.9% (72/106), 55.4% (123/222), and 46.2% (54/107) in the 4 sets, respectively.

**Table 1.** Clinicopathological characteristics in the training, internal test, external test, and prospective test sets.

Characteristics	Training set	Internal test set	External test set	Prospective test set
ALNM				
Negative	149 (35.4)	34 (32.1)	99 (44.6)	63 (53.8)
Positive	272 (64.6)	72 (67.9)	123 (55.4)	54 (46.2)
Age, mean ± SD, years	54.0 ± 10.6	52.5 ± 8.8	54.4 ± 11.6	53.8 ± 10.0
Tumor size, cm	2.9 ± 1.5	2.7 ± 1.1	2.9 ± 1.5	2.4 ± 1.0
Tumor location				
Right	194 (46.1)	54 (50.9)	102 (45.9)	58 (49.6)
Left	227 (53.9)	52 (49.1)	120 (54.1)	59 (50.4)
Orientation				
Parallel	360 (85.5)	100 (94.3)	190 (85.6)	106 (90.6)
Nonparallel	61 (14.5)	6 (5.7)	32 (14.4)	11 (9.4)
Margin				
Well-circumscribed	61 (14.5)	23 (21.7)	54 (24.3)	26 (22.2)
Non-circumscribed	360 (85.5)	83 (78.3)	168 (75.7)	91 (77.8)
Shape				
Oval or round	11 (2.6)	4 (3.8)	8 (3.6)	4 (3.4)
Irregular	410 (97.4)	102 (96.2)	214 (96.4)	113 (96.6)
Echotexture				
Hypoechoic	349 (82.9)	95 (89.6)	188 (84.7)	85 (72.6)
Heterogeneous	72 (17.1)	11 (10.4)	34 (15.3)	32 (27.4)
Microcalcification				
Absent	166 (39.4)	34 (32.1)	110 (49.5)	47 (40.2)
Present	255 (60.6)	72 (67.9)	112 (50.5)	70 (59.8)
Posterior features				
None	223 (53.0)	46 (43.4)	90 (40.5)	47 (40.2)
Shadowing	151 (35.9)	41 (38.7)	103 (46.4)	50 (42.7)
Enhancement	47 (11.1)	19 (17.9)	29 (13.1)	20 (17.1)
Vascularity				
Adler grading 0	55 (13.1)	14 (13.2)	27 (12.2)	10 (8.5)
Adler grading 1	198 (47.0)	57 (53.8)	121 (54.5)	69 (59.0)
Adler grading 2	118 (28.0)	29 (27.4)	50 (22.5)	27 (23.1)
Adler grading 3	50 (11.9)	6 (5.7)	24 (10.8)	11 (9.4)
US-reported ALNM				
Absent	155 (36.8)	24 (22.6)	129 (58.1)	60 (51.3)
Present	266 (63.2)	82 (77.4)	93 (41.9)	57 (48.7)
ER status				
Negative	147 (34.9)	32 (30.2)	78 (35.1)	32 (27.4)
Positive	274 (65.1)	74 (69.8)	144 (64.9)	85 (72.6)
PR status				
Negative	147 (34.9)	38 (35.8)	107 (48.2)	33 (28.2)
Positive	274 (65.1)	68 (64.2)	115 (51.8)	84 (71.8)
HER2 status				
Negative	267 (63.4)	68 (64.2)	161 (72.5)	88 (75.2)
Positive	154 (36.6)	38 (35.8)	61 (27.5)	29 (24.8)
Ki-67 status				
<20%	127 (30.2)	29 (27.4)	86 (38.7)	29 (24.8)
≥20%	294 (69.8)	77 (72.6)	136 (61.3)	88 (75.2)
Molecular type				
Luminal A	91 (21.6)	21 (19.8)	62 (27.9)	27 (23.1)
Luminal B	210 (49.9)	58 (54.7)	87 (39.2)	64 (54.7)
HER2 positive	63 (15.0)	16 (15.1)	36 (16.2)	13 (11.1)
Triple negative	57 (13.5)	11 (10.4)	37 (16.7)	13 (11.1)
Histologic type				
Non-special type invasive breast cancer	408 (96.9)	102 (96.2)	211 (95.0)	102 (95.3)
Others	13 (3.1)	4 (3.8)	11 (5.0)	5 (4.7)

Abbreviations: ALNM, axillary lymph node metastasis; US, ultrasound.

In the training set, univariate analysis revealed several clinical-ultrasonic factors that were significantly different between the ALN + and ALN- groups, including tumor size, margin, microcalcifications, and US-reported ALNM (Table 2). Next, we used multivariate logistic regression to develop a clinical model assessing ALNM using these identified risk factors. Microcalcification was excluded from the model due to its lack of a significant association in the multivariate analysis. The clinical model yielded AUC values of 0.78, 0.79, 0.73, and 0.75 for the training, internal test, external test, and prospective test sets, respectively.

**Table 2.** Results of the univariate and multivariate logistic regression analysis in the training set.

Characteristic	Univariate analysis		Multivariate analysis			
			Clinical model		Clinic-radiomics model	
	OR (95% CI)	*P* value	OR (95% CI)	*P* value	OR (95% CI)	*P* value
Age	0.99 (0.97, 1.01)	.47	NA	NA	NA	NA
Tumor size	1.75 (1.44, 2.12)	<.01*	1.52 (1.24, 1.87)	<.01*	NA	NA
Tumor location (Right)	0.99 (0.66, 1.47)	.94	NA	NA	NA	NA
Orientation (Nonparallel)	0.65 (0.37, 1.12)	.12	NA	NA	NA	NA
Margin (Non-circumscribed)	2.90 (1.67, 5.05)	<.01*	3.27 (1.75, 6.12)	<.01*	NA	NA
Shape (Irregular)	2.24 (0.67, 7.47)	.19	NA	NA	NA	NA
Echotexture (Heterogeneous)	1.29 (0.76, 2.17)	.34	NA	NA	NA	NA
Microcalcification (Present)	1.62 (0.82, 1.79)	.02*	NA	NA	NA	NA
Posterior features						
None	Reference					
Shadowing	1.28 (0.82, 1.99)	.28	NA	NA	NA	NA
Enhancement	0.58 (0.31, 1.10)	.10	NA	NA	NA	NA
Vascularity						
Adler grading 0	Reference					
Adler grading 1	0.88 (0.47, 1.63)	.68	NA	NA	NA	NA
Adler grading 2	1.36 (0.69, 2.67)	.38	NA	NA	NA	NA
Adler grading 3	1.21 (0.54, 2.73)	.64	NA	NA	NA	NA
US-reported ALNM (Present)	5.91 (3.81, 9.16)	<.01*	4.68 (2.94, 7.46)	<.01*	2.24 (1.19, 4.21)	.04*
Tumor score	3.78 (2.83, 5.06)	<.01*	NA	NA	2.72 (1.90, 3.90)	<.01*
LN score	3.76 (2.90, 4.86)	<.01*	NA	NA	3.14 (2.39, 4.11)	<.01*

Abbreviations: ALNM, axillary lymph node metastasis; LN, lymph node; OR, odds ratio; US, ultrasound. *P < .05.

### Feature selection and score building

From each ROI within the primary tumor and LN images, a total of 851 handcrafted features and 128 deep-learning features were extracted. After assessing the ICC and standardizing the features, we conducted a redundancy analysis followed by the least absolute shrinkage and selection operator regression to select the most relevant features. As illustrated in **Figure S2**, least absolute shrinkage and selection operator regression identified 14 features from the tumor and 9 from the LN, which were used to develop the tumor score and LN score, respectively. **Supplementary Material** contains comprehensive details about the feature selection process and the formulas used to calculate the tumor and LN scores.

Univariate analysis revealed a significant association between both the tumor and LN scores and ALNM. The AUCs for the tumor score and LN score in predicting ALNM were 0.80 and 0.91 in the training set, 0.76 and 0.85 in the internal test set, 0.76 and 0.88 in the external test set, and 0.81 and 0.91 in the prospective test set, respectively.

### Construction and evaluation of the clinical-radiomics Model

**Figure 1.**
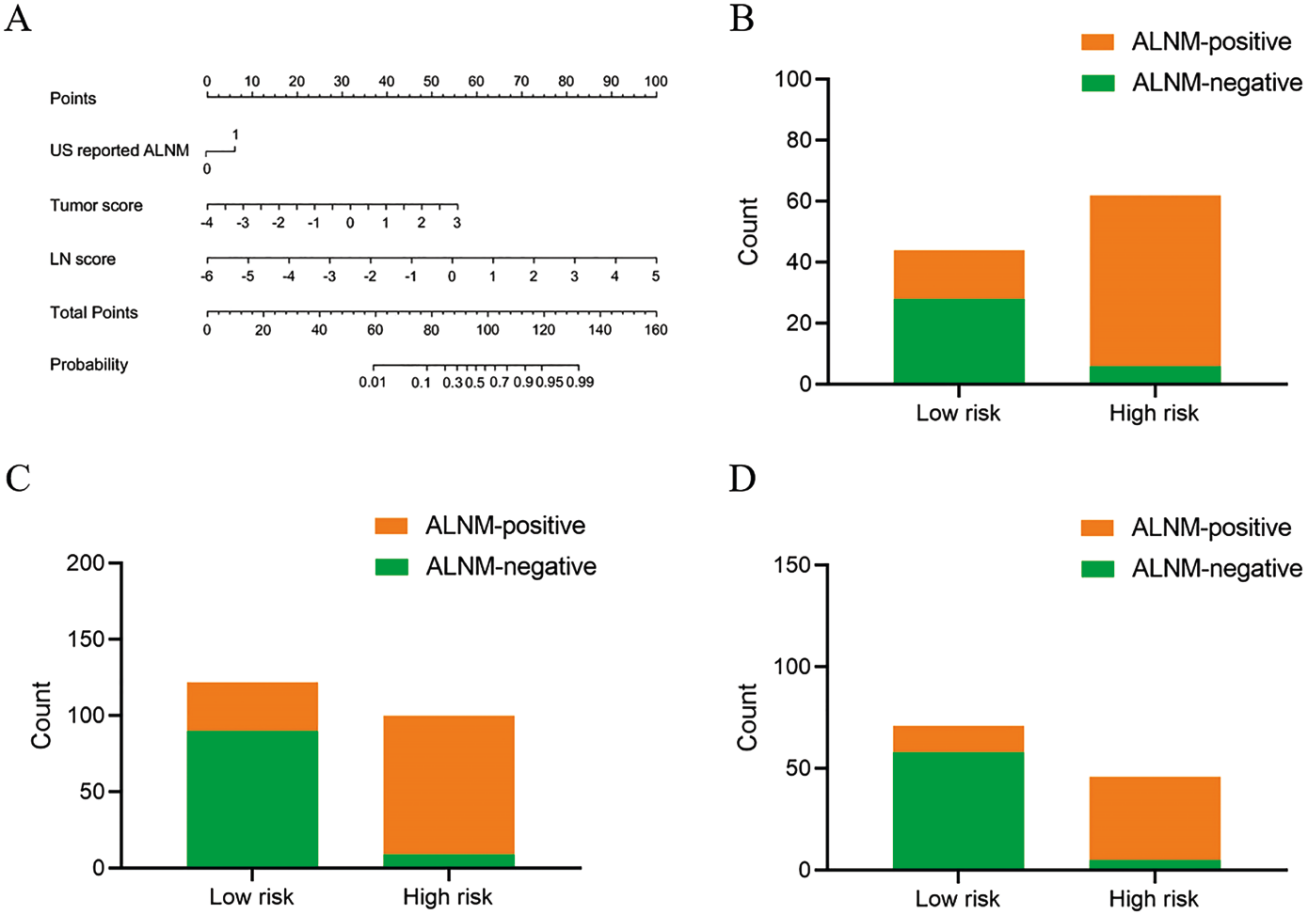
Development and performance of the clinic-radiomics model. (A) Nomogram for predicting the probability of ALNM. (B-D) The risk-classification performance of the clinic-radiomics model in the internal (B), external (C) and prospective test set (D), respectively. Abbreviations: ALNM, axillary lymph node metastasis; LN, lymph node; US, ultrasound.

Multivariate analysis identified US-reported ALNM, tumor score, and LN score as independent predictive factors of ALN status (**Table 2**). These variables were used to construct the clinical-radiomics model shown in **Figure 1A**. According to the Hosmer-Lemeshow test, the model demonstrated good calibration for assessing ALN status across all 4 sets, with *P*-values of .17, .28, .27, and .76, respectively. The ideal cutoff value for evaluating ALNM using the Nomo-score, determined by the Youden index, was found to be 0.701. **Table 3** illustrates the performance of the model at this threshold. The model achieved AUC values of 0.94 for the training set, 0.92 for the internal test set, 0.91 for the external test set, and 0.95 for the prospective test set.

**Table 3.** Performance of different models for evaluating ALN status.

	AUC (95% CI)	ACC	SEN	SPE	PPV	NPV
Training set						
Clinical model	0.78 (0.74-0.82)	72.9%	80.5%	68.5%	82.3%	65.8%
Tumor score	0.80 (0.76-0.84)	80.5%	90.8%	62.4%	81.5%	78.8%
LN score	0.91 (0.87-0.93)	82.4%	82.7%	85.2%	91.1%	73.0%
Clinic-radiomics model	0.94 (0.91-0.96)	86.5%	86.4%	89.9%	94.0%	78.4%
Internal test set						
Clinical model	0.79 (0.70-0.86)	79.2%	90.3%	55.9%	81.3%	73.1%
Tumor score	0.76 (0.67-0.84)	71.7%	87.5%	38.2%	75.0%	59.1%
LN score	0.83 (0.77-0.88)	73.6%	70.8%	79.4%	87.9%	56.3%
Clinic-radiomics model	0.92 (0.85-0.96)	79.2%	77.8%	82.4%	90.3%	63.6%
External test set						
Clinical model	0.73 (0.67-0.79)	68.5%	60.2%	78.8%	77.9%	61.4%
Tumor score	0.76 (0.70-0.82)	68.0%	67.5%	68.7%	72.8%	63.0%
LN score	0.88 (0.83-0.92)	76.1%	64.2%	90.9%	89.8%	67.2%
Clinic-radiomics model	0.91 (0.87-0.95)	81.5%	74.0%	90.9%	91.0%	73.8%
Prospective test set						
Clinical model	0.75 (0.66-0.82)	68.4%	66.7%	69.8%	65.5%	71.0%
Tumor score	0.81 (0.73-0.88)	61.5%	100.0%	28.6%	54.5%	100.0%
LN score	0.91 (0.85-0.96)	63.2%	22.2%	98.4%	92.3%	59.6%
Clinic-radiomics model	0.95 (0.89-0.98)	84.6%	75.9%	92.1%	89.1%	81.7%

Abbreviations: Acc, accuracy; AUC, area under the receiver operating characteristic curve; CI, confidence interval; LN, lymph node; NPV, negative predictive value; PPV, positive predictive value; SEN, sensitivity; SPE, specificity.

We performed further evaluations of the clinical-radiomics model’s discriminative ability in predicting ALNM across the internal, external, and prospective test sets. Each test set was stratified based on the Nomo-score, with subsets identified as high-risk and low-risk. The results indicated that the high-risk group in each cohort had a greater proportion of ALNM cases (**Figure 1**). Furthermore, according to clinical impact curve analysis, when the probability thresholds surpassed 75%, 70%, and 65% in the internal, external, and prospective test sets, respectively, the number of high-risk individuals closely matched those with actual ALNM, indicating a high level of clinical predictive efficacy (**Figure S3**).

**Figure 2.**
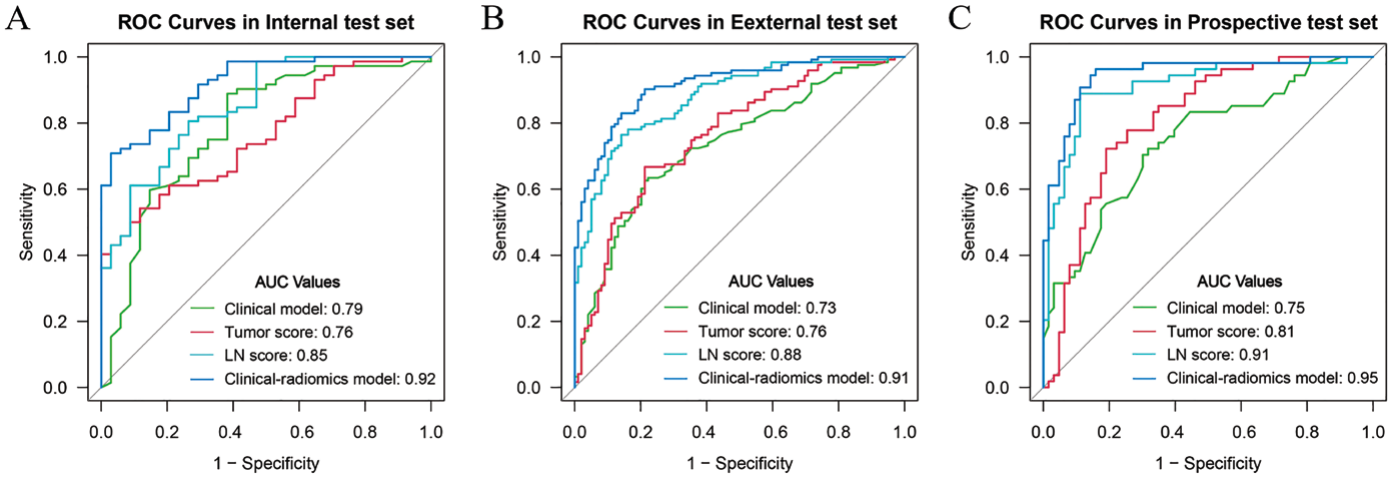
The receiver operating characteristic curves for the prediction of the ALN status in the internal (A), external (B) and prospective test set (C).

**Figure 2** displays the receiver operating characteristic curves for the various models. According to the DeLong test (**Table S3**), the clinical-radiomics model demonstrated statistically superior performance compared to the clinical model, tumor score and LN score in all 3 test sets (all *P* < .05). The DCA curves indicated that the clinical-radiomics model yielded greater net benefit for ALNM assessment compared to the clinical model and single score across varying threshold probability ranges: 0.4 to 0.94 in the internal test set, 0.18 to 0.94 in the external test set, and 0.04 to 0.43 and 0.47 to 0.8 in the prospective test set (**Figure S4**). Furthermore, in contrast to the clinical model that solely incorporated clinical-ultrasonic factors, integrating tumor score and LN score significantly improved the predictive effectiveness of the clinical-radiomics model for ALNM. This improvement is evidenced by substantial improvements in NRI and IDI parameters across all 3 test sets (**Table S4**).

### Radiologist assessment and AI-assisted assessment

**Figure 3.**
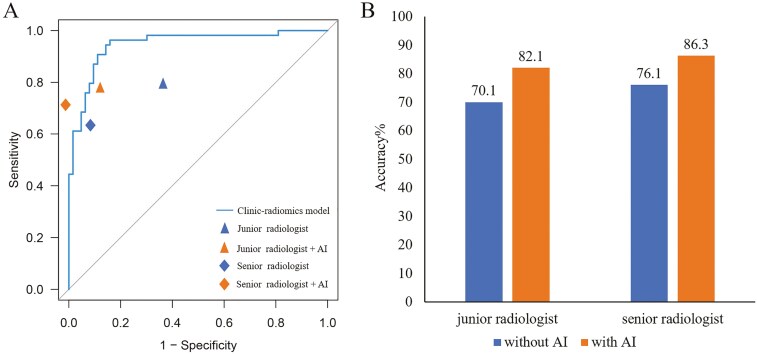
The ROC plots of the clinic-radiomics model and radiologists without and with artificial intelligence (AI) assistance in the prospective test set (A). Accuracy with or without AI-assisted diagnosis in the prospective test set (B).

As detailed in **Tables 3** and **Table S5**, the clinical-radiomics model exhibited superior ability in predicting ALNM compared to initial diagnoses by junior and senior radiologists in the prospective test set (AUC: 0.95 vs 0.71 and 0.75, respectively). The integration of the clinical-radiomics model notably enhanced radiologists’ diagnostic performance, increasing AUC to 0.82 for the junior radiologist and 0.85 for the senior radiologist (**Figure 3A).** The accuracy rates for the junior and senior radiologists improved significantly from 70.1% to 82.1% and from 76.1% to 86.3%, respectively (**Figure 3B).** AI assistance also markedly increased the specificities for junior radiologists from 61.9% to 85.7% and for senior radiologists from 87.3% to 98.4%. Furthermore, with AI support, the Kappa values for both radiologists in the prospective test set increased from 0.557 to 0.733.

## Discussion

In this multicenter study, we developed and validated a clinical-radiomics model that combines clinical-ultrasonic factors with tumor and LN radiomics signatures to assess ALNM preoperatively in breast cancer patients. Additionally, we investigated whether this model could enhance radiologists’ diagnostic accuracy. To the best of our knowledge, this research represents the first attempt to utilize a deep-learning radiomics method using tumor and LN US images to evaluate ALNM in breast cancer.

The status of ALN is crucial for guiding clinical treatment and prognostic evaluation.^22^ Previous studies have shown that US-reported tumor size and axillary US findings are correlated with ALN status in breast cancer. However, the relatively low AUC values of 0.59-0.72 reported in these studies highlight the challenge that radiologists face in accurately predicting ALNM.^20,23,24^ Some studies have attempted to predict ALN status using pathological data, such as lymphovascular invasion, Ki-67 proliferation index, and molecular subtype.^25-27^ However, reliance on pathological data alone is not sufficiently accurate. Additionally, some factors, such as lymphovascular invasion, are not available preoperatively. Since knowing ALN status before surgery is crucial for determining appropriate axillary treatment options. Unlike previous studies, this research utilized preoperatively accessible clinical US information as candidate variables for model development, offering a noninvasive method for assessing ALN status.

Radiomics is an emerging technology that transforms medical images into high-throughput features.^28^ These features, such as intensity, wavelet, or texture, provide information about the tumor microenvironment that cannot be discerned by radiologists and offer complementary information to clinically obtained or treatment-related data.^29,30^ However, most previous radiomics studies on ALNM have mainly focused on the radiomics characteristics of the primary breast tumor, neglecting the significance of the ALN.^25,31-33^ According to the “seed and soil” hypothesis, the initiation of ALNM depends on the interactive relationship between tumor cells (seed) and the ALN microenvironment (soil).^5^ Tumor cells exhibit a specific affinity for specific organs or tissues, and metastasis occurs when this match between the seed and the soil is established. Given the connection between ALN and the primary tumor, this study delineated the ROI of both the primary tumor and ALN, capturing complementary biological information. Tumor features reflect its aggressiveness (eg, heterogeneity, invasiveness), while ALN features reveal the microenvironment’s receptiveness to metastasis. This integration aligns with the “seed and soil” theory of cancer metastasis. Furthermore, many previous radiomics-based ALNM prediction studies have been limited by small sample sizes or single-center data, lacking robustness and generalizability.^11,16,21,32,33^ The multicenter validation in our study indicates that the US radiomics score, developed using various types of equipment, is broadly applicable and reproducible across all 3 test sets for predicting ALNM.

In addition to constructing the radiomics score, we incorporated easily accessible preoperative clinical-ultrasonic risk factors and developed a deep-learning radiomics model based on multivariate analysis. To facilitate clinical application, we visualized the model as a nomogram, providing radiologists with an intuitive and effective tool for evaluating the ALN status. The deep learning radiomics model exhibited excellent and robust discriminative performance across the internal, external, and prospective test sets, with AUCs of 0.92, 0.91, and 0.95, respectively. This performance surpasses that of the clinical model, as well as the single radiomics score. Additionally, significant improvements in NRI and IDI showed that the combination of tumor and LN scores substantially enhanced the model’s performance in predicting ALNM. These 2 scores could serve as novel indicators for evaluating ALNM. The DCA curves further illustrated that using the deep learning radiomics model to predict ALNM provided a superior overall net benefit compared to the clinical model, single score, and the “treat all” or “treat none” approaches across most threshold probabilities.

Although various studies have developed effective AI models for evaluating ALNM in breast cancer, their application in clinical settings has yet to be confirmed.^34-36^ In our prospective test set, we employed a 2-step US review process to evaluate whether AI assistance could enhance radiologists’ interpretations. The first diagnosis was based solely on the radiologists’ experience, whereas the second diagnosis utilized predictions from the AI model. Incorporating the AI model led to a notable enhancement in both AUC and accuracy for the second diagnosis, highlighting the AI’s capability to identify potential tumor heterogeneity that may be missed during the initial US assessment. In cases where there was a significant discrepancy between the AI model’s result and the radiologist’s diagnosis, it encouraged a more thorough evaluation, resulting in improved diagnostic accuracy.

Our study has several limitations. First, patients with multifocal and bilateral breast lesions were excluded due to the difficulty in identifying which lesion might lead to ALNM. However, the excellent performance of our dual-region approach establishes a reliable framework for future extensions. We are actively planning follow-up studies to address multifocal and bilateral cases by developing lesion-specific radiomics signatures, incorporating spatial relationships between lesions and LNs, as well as collaborating with pathologists to identify molecular markers linking specific lesions to ALNM. Second, the small number of radiologists involved may not fully reflect the capabilities of a broader radiological workforce. Future research should include a larger cohort of radiologists to more thoroughly assess the model’s auxiliary effectiveness. Third, this study relies on the manual delineation of ROIs, which may limit the applicability of the method in routine clinical practice. Automated tumor segmentation is an important direction for future development to enhance efficiency and clinical applicability. Lastly, our multicenter study comprised participants exclusively from China. To better evaluate the model’s generalizability, it is necessary to test it with larger datasets from diverse regions and countries. Additionally, extending the prospective cohort follow-up to assess the model’s impact on patient outcomes, such as reducing unnecessary surgeries or improving survival, would provide valuable insights. This remains an important direction for future research.

## Conclusion

In conclusion, our study illustrates the feasibility and effectiveness of using deep-learning radiomic features from breast tumors and LN US images to construct a predictive model for ALNM in breast cancer. This noninvasive approach holds significant potential to improve preoperative assessment and guide clinical decision-making, ultimately enhancing patient outcomes and optimizing resources in breast cancer management.

## Supplementary Material

oyaf090_suppl_Supplementary_Material

oyaf090_suppl_Supplementary_Figures_S1-S5

oyaf090_suppl_Supplementary_Tables_S1-S5

## Data Availability

The code and datasets used in this study will be made available upon request to facilitate reproducibility and further research. Researchers interested in accessing the data or code may contact the corresponding author for details.
