# Supplementary material for: Ultrasound-based deep learning radiomics for enhanced axillary lymph node metastasis assessment: a multicenter study
Source: Oncologist. 2025 May 11;30(5):oyaf090. doi: 10.1093/oncolo/oyaf090 (PMC12065944; doi:10.1093/oncolo/oyaf090)
Supplement: oyaf090_suppl_Supplementary_Material [file oyaf090_suppl_supplementary_material.docx]

**Supplemental S1. Multicenter selection rationale and patient inclusion/exclusion criteria**

1. Multicenter selection rationale:

(1) All institutions are tertiary hospitals in China, ensuring a high level of expertise and a sufficient number of patient examinations; (2) accredited breast ultrasound departments; (3) adoption of the Breast Imaging Reporting and Data System (BI-RADS) for standardized reporting; (4) established standardized histopathology reporting systems.

2. Patient inclusion/exclusion criteria:

For the retrospective study, the inclusion criteria were as follows: (1) all patients underwent breast US examination within two weeks prior to surgery; (2) histologically confirmed primary invasive breast cancer; (3) ALN status was confirmed through SLNB or ALND; Exclusion criteria included (1) preoperative neoadjuvant radiotherapy or chemotherapy before US examination; (2) multifocal lesions or bilateral disease; (3) missing clinicopathological data; (4) incomplete or poor-quality images.

For the prospective study, the inclusion criteria were as follows: (1) women with suspicious breast lesions after US examination; (2) patients planned to undergo biopsy or surgery; (3) patients undergoing SLNB or ALND within 2 weeks after the US examination; (4) availability of clinical data. The exclusion criteria included (1) non-primary invasive breast cancer, such as benign breast lesions or carcinoma in situ; (2) preoperative neoadjuvant radiotherapy or chemotherapy; (3) multifocal lesions or bilateral disease; (4) missing important histopathological results, such as immunohistochemical results or postoperative pathological ALN status; (5) incomplete information or imaging data.

**Supplemental S2. US examination process and ultrasonic features evaluation**

Breast US images were primarily obtained using a range of equipment, including Resona 5S, Resona 6S, Resona 7S, Resona 7T, Resona 8, DC-8 and Nuewa R9 from MINDRAY Corporation, GE VOLUSON E8, Philips iU22, Philips EPIQ5, Philips EPIQ7, Philips EPIQ7C, SAMSUNG WS80A, SAMSUNG RS80A, SIMENS ACUSON S2000, Siemens ACUSON Sequoia, SuperSonic Imaging AixPlorer, Esaote MyLab 9, HITACHI ARIETTA 70 and GE LOGIQ E9, all equipped with high-frequency linear probes. The examinations were conducted by board-certified radiologists with more than three years of experience in performing and interpreting breast ultrasound across six hospitals. Variations existed in the ultrasound machines utilized across different hospital settings. All patients were examined in a supine position with their arms abducted to ensure full exposure of both breasts. Optimal settings for depth, gain, and focal zones were carefully adjusted to ensure the acquisition of high-quality ultrasound images and the maximum diameter of each lesion was recorded. The status of ALN was assessed in the process of US examination. The images containing important features of primary tumor and ALN were stored into the Picture Archiving and Communication Systems (PACS) for subsequent analysis and validation.

Two radiologists, each with over 15 years of experience in breast US, independently conducted blinded reviews of all US images. They assessed the US features of each patient according to the Breast Imaging Reporting and Data System lexicon[1]. Any discrepancies were resolved by a consensus decision. The evaluated US features included lesion orientation, margin, shape, echotexture, microcalcifications, posterior characteristics (lesions with mixed posterior features were categorized based on the predominant component).

Blood flow in breast tumors was evaluated using the Adler semi-quantitative grading system[2,3] , which classifies blood flow signals as follows: Adler 0 indicates no detectable blood flow signals; Adler I represents one or two punctate or short blood flow signals; Adler II includes three or four punctate or short signals, or one longer signal; and Adler III encompasses more than five punctate or short signals, or two longer signals, or the presence of intertwined blood vessels forming a network.

**Supplemental S3. Radiomics feature extraction**

Prior to feature extraction, we applied uniform preprocessing to the images. For the radiomics feature extraction, the following steps were implemented: (1) resampling: to ensure consistency in spatial resolution and size across images from different sources or time points, we applied resampling techniques, thereby eliminating any disparities caused by variations in equipment or temporal factors. (2) standardization: all images were adjusted to the same resolution and size, ensuring consistent spatial resolution and minimizing biases introduced by different devices or operators. (3) normalization: to achieve comparability in gray-level distribution across images from different devices or taken at different times, we normalized the contrast and brightness of the images, ensuring uniformity in their gray-scale distribution. The initially extracted features comprise two components:

**Section 1: Deep Learning Feature Extraction**

The VGG19 architecture has shown exceptional performance in various image processing tasks, such as image classification, object detection, and semantic segmentation[4,5]. One of the key attributes of VGG19 is its simple and consistent design. It comprises 19 weight layers, which include both convolutional and fully connected layers. The architecture employs a pattern where pairs of consecutive 3x3 convolutional filters are succeeded by a 2x2 max-pooling layer, with this sequence repeating to create convolutional blocks. This design enables VGG19 to efficiently extract image features while preserving its structural simplicity.

Due to the limited size of the dataset, assembling images from scratch and training domain-specific classifiers is considerably challenging. This study primarily focuses on the task of image classification. To enhance classification performance, a transfer learning strategy was employed. During the deep learning feature extraction process, a deep convolutional neural network (DCNN) was initialized using pretrained weights from ImageNet, a dataset containing over 12 million natural images across 1000 categories. The pretrained VGG19 model was subsequently fine-tuned to suit the specific classification task.

By modifying the architecture of VGG19, DCNNs were designed by removing the top layers, retaining the pretrained model weights, and adding new top layers for training. Numerous 2D images of ROIs from the training set were utilized for training to predict ALNM. For feature extraction, ultrasound images were input into the hierarchical convolutional framework of the DCNNs. Multiple feature maps were extracted and selected from the activation layer of the final convolutional layer, which has 128 dimensions. These feature maps were then quantified to derive their statistical properties, which served as deep learning features. Deep learning feature extraction was performed on the ROIs of both tumor and LN images. The implementation details are as follows.

Before feeding the images into the deep learning network, Radiologist A performed segmentation and masking on all US images. Initially, the size of the square segmentation mask was adjusted to ensure adequate edge spacing between the lesion boundary and the mask’s boundary, without exceeding the sampling frame. The tumor and LN images were then cropped to focus on the lesion area. Radiologist B then refined the image range for each mask to ensure the lesion and mask proportions were consistent. To incorporate maximum lesion information, the smallest ROI was carefully selected. These adjusted images served as input for the deep neural network. Upon importing the images, an automatic scaling process resized them to a standardized 224×224 dimension.

The DCNNs were optimized using the stochastic gradient descent algorithm with mean squared error as the loss function. Each mini-batch contained 16 image patches. In the initial phase, the convolutional layers were frozen, and only the final layer was trained with a small learning rate of 0.001. In the subsequent phase, we trained all layers, further reducing the initial learning rate to one-tenth of the original rate. To mitigate overfitting and improve the convolutional layers’ representational capabilities, L2 regularization was applied to the final layer. For learning rate adjustment and early stopping, 20% of patients from the training set were randomly selected to assess validation performance. The learning rate was reduced by a factor of 10 if it did not improve after 5 epochs, and early stopping was activated if no improvement was seen after 10 epochs. In total, 128 deep learning features were extracted from each ROI. Our DCNNs were implemented using the Python Keras package (https://github.com/fchollet/keras) in conjunction with the TensorFlow library (<https://www.tensorflow.org>).

**Section 2: Hand-crafted feature extraction**

The hand-crafted radiomics feature extraction algorithms were standardized in accordance with the Image Biomarker Standardization Initiative (IBSI) guidelines[6]. Features were extracted using the "pyradiomics" package in Python (version 3.10.6). Each ROI, along with its corresponding tumor and LN images, yielded a total of 851 quantitative imaging features. These included 14 shape features, 162 first-order features, 216 gray level co-occurrence matrix (GLCM) features, 126 gray level dependence matrix (GLDM) features, 144 gray level run length matrix (GLRLM) features, 144 gray level size zone matrix (GLSZM) features, and 45 neighboring gray tone difference matrix (NGTDM) features. For further details on feature extraction methods, image types, and parameter settings, refer to the radiomics documentation (https://pyradiomics.readthedocs.io/en/latest).

**Supplemental S4. Radiomics feature selection and score building process**

The feature selection and score construction process was performed on both ROIs of tumor and LN including three steps: a) assessing feature reproducibility through inter-observer and intra-observer agreement, b) retaining a comprehensive set of representative features with mutual information, and c) constructing scores. The detailed descriptions are as follows:

First, intra- and inter-observer variability was evaluated, and radiomics features with an ICC < 0.80 were excluded. After merging the remaining hand-crafted and deep learning features, the feature distributions in the training, internal test, external test, and prospective test sets were standardized using the Z-score transform. This normalization ensured that different data were on the same scale, reducing the impact of varying protocols and operators on the US images and making the radiomics features comparable. Additionally, redundancy analysis was performed on the extracted features from the training set. Normality testing was conducted, with Pearson correlation analysis applied to normally distributed features and Spearman correlation analysis used for non-normally distributed features.

Redundant tumor and LN features with a correlation coefficient greater than 0.85 were removed. To reduce overfitting or selection bias in the radiomics column maps, the least absolute shrinkage and selection operator (LASSO) regression was used to select the remaining features. Five-fold cross-validation was used to determine the optimal penalty coefficient (lambda). The radiomics features with non-zero coefficients in the training set were obtained, and these non-zero coefficients were weighted to create formulas for the tumor and LN scores, respectively. By following these steps, we established the tumor score, representing tumor features, and the LN score, representing LN features, as predictive factors for ALNM.

**Supplemental S5. The process of feature selection and the calculation formula for tumor and LN scores.**

Feature selection was carried out using R software. Initially, 851 handcrafted features and 128 deep learning features were extracted from each ROI, along with their corresponding tumor and LN images. To ensure the stability and reliability of the features used in the final model, the ICC of both intra-observer and inter-observer variability was assessed. After random sampling for ICC analysis, features with an intra-observer or inter-observer ICC < 0.80 were removed, leaving 488 tumor handcrafted features and 499 LN handcrafted features. These remaining handcrafted features were then combined with the deep learning features and standardized. After conducting redundancy analysis, 20 tumor features and 61 LN features were selected for LASSO regression. The penalty coefficient Log(λ) in the LASSO model was determined through 5-fold cross-validation based on the minimum criterion. As λ increased, the coefficients of each feature were incrementally compressed to zero. The optimal value of λ was chosen based on the smallest cross-validation error. Ultimately, 14 tumor features and 9 LN features with non-zero coefficients were identified for constructing the tumor and LN scores, respectively. To clarify, in our feature selection process using LASSO regression, we determined the importance of each feature based on its corresponding coefficient in the model. Features with non-zero coefficients were considered the most significant and were included in the final model. The LASSO method works by shrinking the coefficients of less relevant features to zero, which naturally highlights the most important ones. In our LASSO regression framework, the size and sign (positive or negative) of the non-zero coefficients indicate the relative importance and the effect (whether it enhances or reduces the outcome) of each selected feature. The absolute values of these coefficients effectively act as implicit weights in the final risk score calculation.

The tumor score and LN score were computed using the following formula:

Tumor score = 0.708 - 0.034 × DL_38 + 0.097 × DL_68 + 0.231 × DL_75 + 0.044 × DL_77 - 0.038 × DL_111+ 0.052 × DL_112 + 0.4110 × DL_122+ 0.046 × DL_124 - 0.295 × DL_127 - 0.239 × original_shape_Elongation

+ 0.077 × wavelet.LLH_firstorder_RobustMeanAbsoluteDeviation

+ 0.327 × wavelet.LLH_glrlm_RunLengthNonUniformity

- 1.121 × wavelet.HHH_glcm_MCC - 0.801 × wavelet.LLL_glszm_ZonePercentage

LN score = 0.821 + 0.185 × DL_37 - 0.061 × DL_40 + 0.433 × DL_47 - 0.187 × DL_107 - 0.346 × DL_120

+ 0.203 × wavelet.LLH_firstorder_RobustMeanAbsoluteDeviation

+ 1.209 × wavelet.HLH_glrlm_LowGrayLevelRunEmphasis

- 0.056 × wavelet.HHH_firstorder_RobustMeanAbsoluteDeviation

+ 0.832 × wavelet.HHH_glszm_ZoneEntropy

**References**

1. D'Orsi C, Morris E, Mendelson E (2013) ACR BI-RADS® Atlas, Breast Imaging Reporting and Data System.

2. Adler DD, Carson PL, Rubin JM, Quinn-Reid D (1990) Doppler ultrasound color flow imaging in the study of breast cancer: preliminary findings. Ultrasound Med Biol 16 (6):553-559. doi:10.1016/0301-5629(90)90020-d

3. Qiu YJ, Cheng J, Zuo D, Zhang Q, Tian XF, Lu XY, Chen S, Dong Y, Wang WP (2023) Non-invasive evaluation of vascular architecture of focal liver lesions by micro vascular imaging. Clin Hemorheol Microcirc 84 (1):43-52. doi:10.3233/ch-221682

4. Khan MA, Rajinikanth V, Satapathy SC, Taniar D, Mohanty JR, Tariq U, Damaševičius R (2021) VGG19 Network Assisted Joint Segmentation and Classification of Lung Nodules in CT Images. Diagnostics (Basel) 11 (12). doi:10.3390/diagnostics11122208

5. Bansal M, Kumar M, Sachdeva M, Mittal A (2023) Transfer learning for image classification using VGG19: Caltech-101 image data set. Journal of ambient intelligence and humanized computing 14 (4):3609-3620. doi:10.1007/s12652-021-03488-z

6. Zwanenburg A, Vallières M, Abdalah MA, Aerts H, Andrearczyk V, Apte A, Ashrafinia S, Bakas S, Beukinga RJ, Boellaard R, Bogowicz M, Boldrini L, Buvat I, Cook GJR, Davatzikos C, Depeursinge A, Desseroit MC, Dinapoli N, Dinh CV, Echegaray S, El Naqa I, Fedorov AY, Gatta R, Gillies RJ, Goh V, Götz M, Guckenberger M, Ha SM, Hatt M, Isensee F, Lambin P, Leger S, Leijenaar RTH, Lenkowicz J, Lippert F, Losnegård A, Maier-Hein KH, Morin O, Müller H, Napel S, Nioche C, Orlhac F, Pati S, Pfaehler EAG, Rahmim A, Rao AUK, Scherer J, Siddique MM, Sijtsema NM, Socarras Fernandez J, Spezi E, Steenbakkers R, Tanadini-Lang S, Thorwarth D, Troost EGC, Upadhaya T, Valentini V, van Dijk LV, van Griethuysen J, van Velden FHP, Whybra P, Richter C, Löck S (2020) The Image Biomarker Standardization Initiative: Standardized Quantitative Radiomics for High-Throughput Image-based Phenotyping. Radiology 295 (2):328-338. doi:10.1148/radiol.2020191145
