# Supplementary material for: Ultrasound-based deep learning radiomics for enhanced axillary lymph node metastasis assessment: a multicenter study
Source: Oncologist. 2025 May 11;30(5):oyaf090. doi: 10.1093/oncolo/oyaf090 (PMC12065944; doi:10.1093/oncolo/oyaf090)
Supplement: oyaf090_suppl_Supplementary_Figures_S1-S5 [file oyaf090_suppl_supplementary_figures_s1-s5.docx]

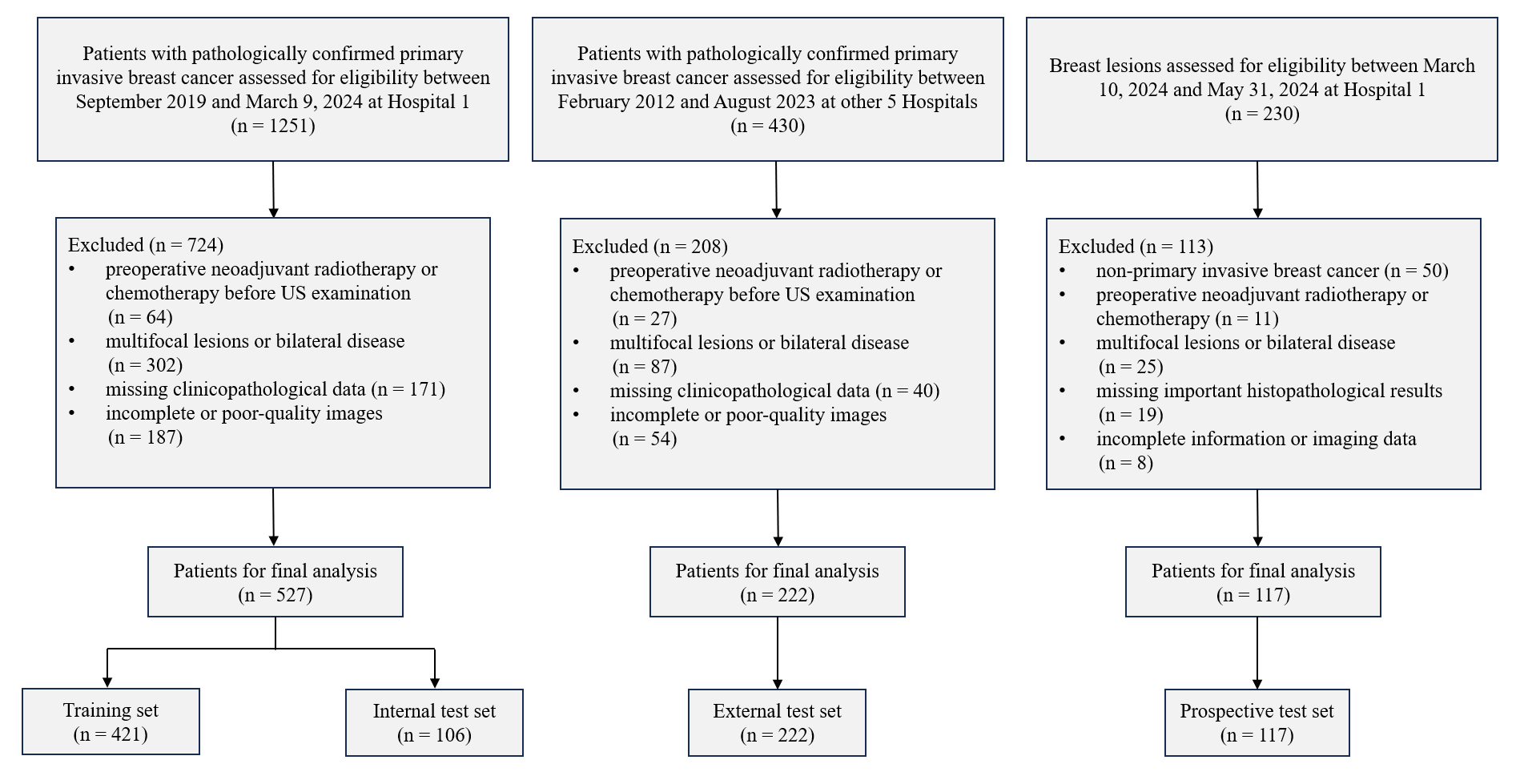


**Supplemental Fig. S1** Flow diagrams of study populations.


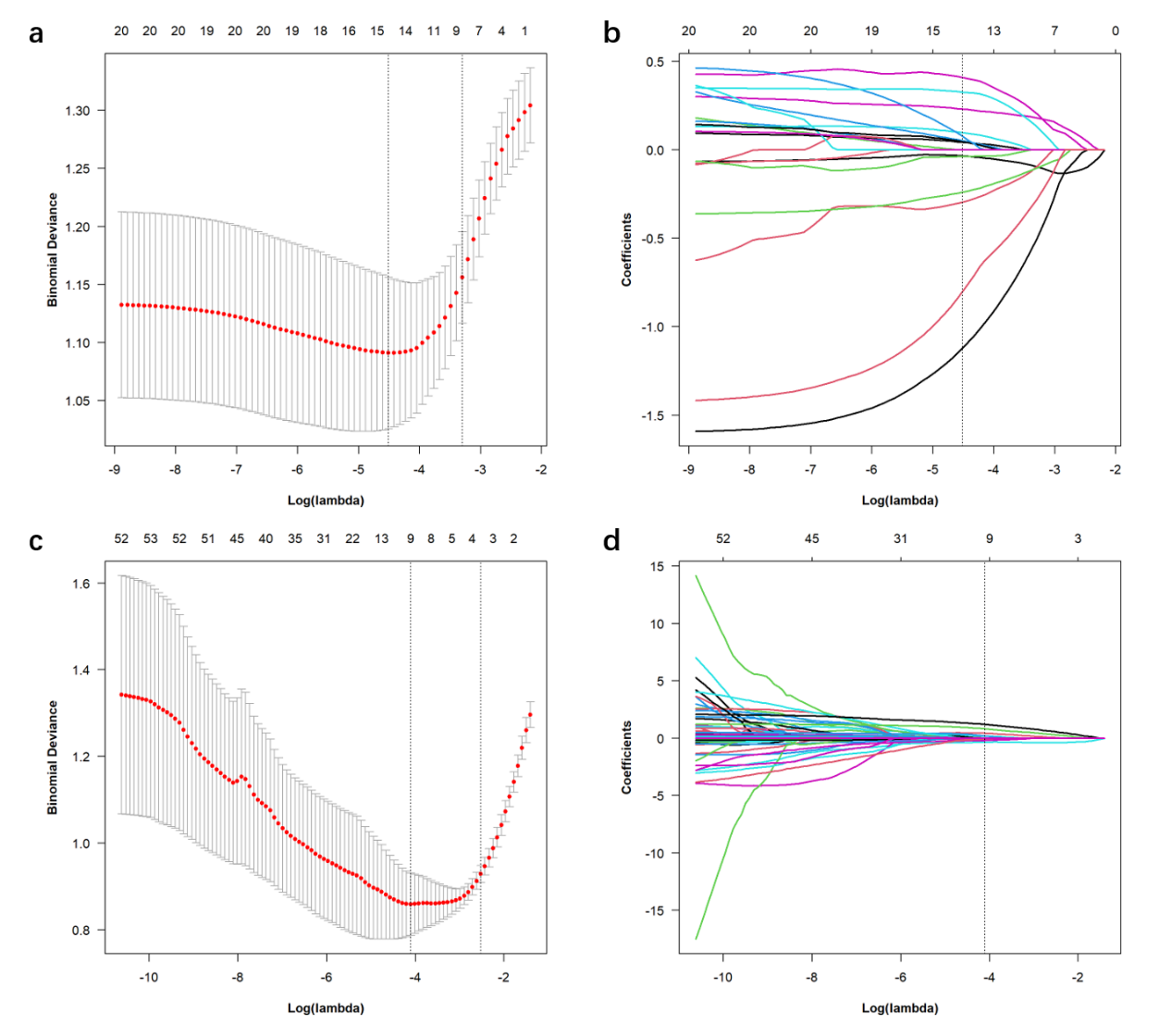


**Supplemental Fig. S2** ALN status-related features selection using the least absolute shrinkage and selection operator (LASSO) logistic regression model in the training set. The five-fold cross-validation and the minimal criteria process was used to generate the optimal penalization coefficient lambda (λ) for the tumor features (a) and LN features (c) in the LASSO model, respectively. LASSO coefficient profiles of the tumor features (b) and LN features (d).


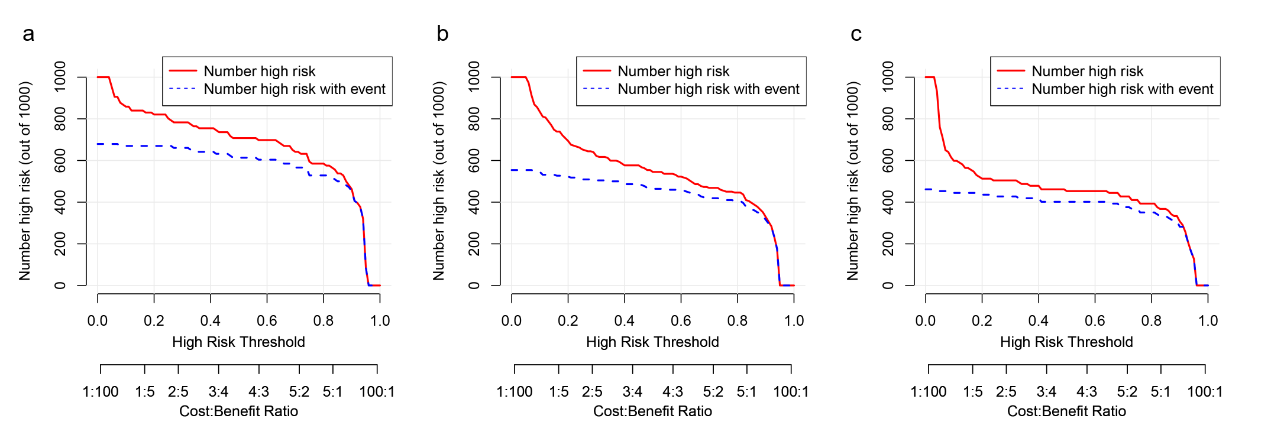


**Supplemental Fig. S3** The clinical impact curve of the clinical-radiomics model at different threshold probabilities in the internal test (c), external test and prospective test set (d).


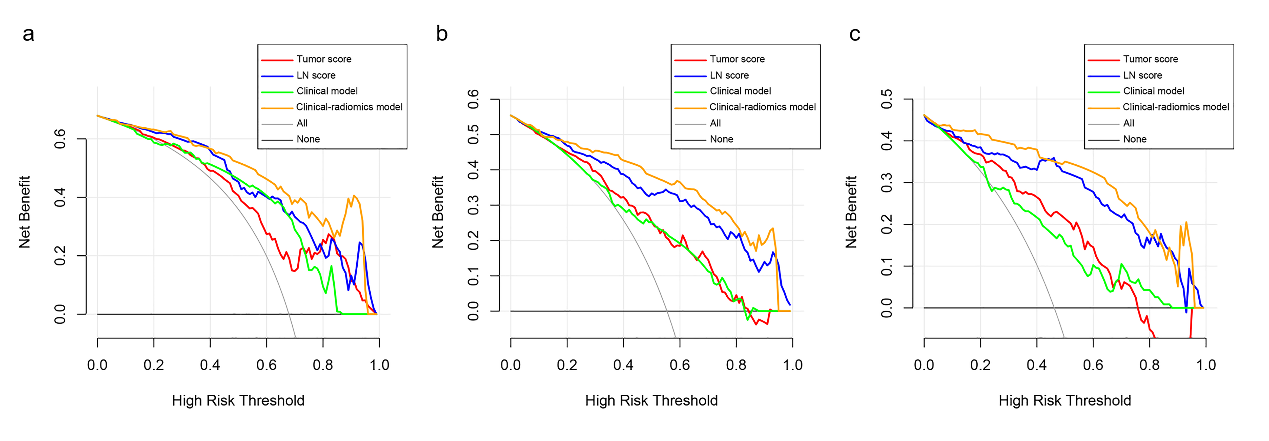


**Supplemental Fig. S4** Decision curve analysis of different models in predicting ALNM derived from the internal (a), external (b) and prospective test set (c).


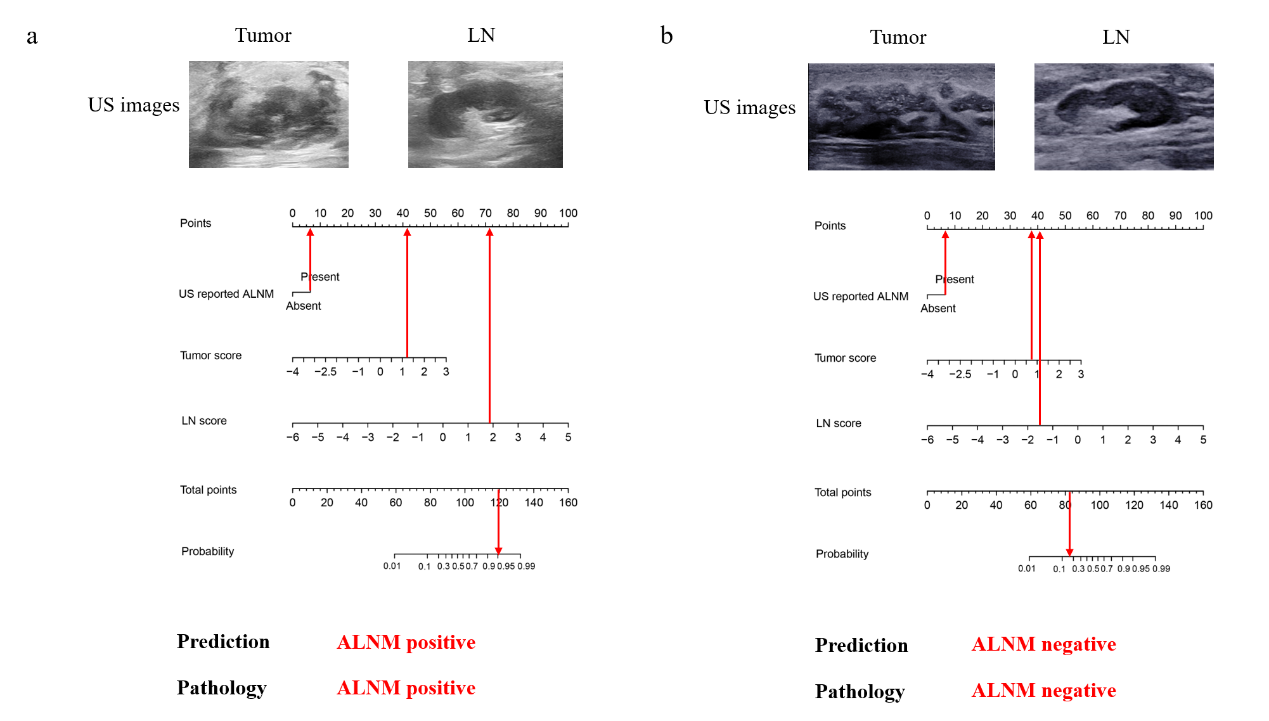


**Supplemental Fig. S5** Diagrams for the clinical application of the nomogram on two representative cases. (a) The breast cancer patient has US-reported ALNM (6 points), with a tumor score of 1.219 (41.5 points) and an LN score of 1.893 (72 points). The total score for this nodule is 119.5, corresponding to a Nomo-score of 0.953, which exceeds the optimal cutoff value of 0.701. Based on the model, this suggests a positive diagnosis of ALNM. The oncologist can now consider the preoperative probability of ALNM positivity, integrating their clinical expertise and the patient’s preferences, to reassess the surgical plan and develop a more aggressive neoadjuvant/adjuvant treatment strategy. (b) The breast cancer patient also has US-reported ALNM (6 points), with a tumor score of 0.733 (37.5 points) and an LN score of -1.493 (40.2 points). The total score for this nodule is 83.7, corresponding to a Nomo-score of 0.161, which is below the optimal cutoff value of 0.701, indicating a negative diagnosis for ALNM. This low-risk patient may benefit from a more conservative surgical approach, a tailored neoadjuvant or adjuvant therapy plan, and less frequent follow-up monitoring.
