# Supplementary material for: Ultrasound-based deep learning radiomics for enhanced axillary lymph node metastasis assessment: a multicenter study
Source: Oncologist. 2025 May 11;30(5):oyaf090. doi: 10.1093/oncolo/oyaf090 (PMC12065944; doi:10.1093/oncolo/oyaf090)
Supplement: oyaf090_suppl_Supplementary_Tables_S1-S5 [file oyaf090_suppl_supplementary_tables_s1-s5.docx]

Supplemental Table S1. Patient sample distribution across participating hospitals.

| Hospital name | Study design | Number of patients |
| --- | --- | --- |
| The First Affiliated Hospital of Anhui Medical University | retrospective and prospective | 527 (retrospective)  107 (prospective) |
| Hefei First People's Hospital | retrospective | 91 |
| Fuyang Cancer Hospital | retrospective | 14 |
| The Second Affiliated Hospital of Anhui Medical University | retrospective | 47 |
| Nanchong Central Hospital | retrospective | 50 |
| Wuhu Hospital Affiliated to East China Normal University | retrospective | 20 |

Supplemental Table S2. Major packages of R software used in this study.

| Functions | R package |
| --- | --- |
| Interclass correlation coefficients (ICC) | psych |
| Standardization of radiomics features | caret |
| Least absolute shrinkage and selection operator (LASSO) regression | glmnet |
| Plot nomogram | rms |
| Draw the receiver operating curve (ROC) and measure the area under the ROC (AUC) | pROC |
| Calibration curves | rms |
| Decision curve analysis (DCA) and clinical impact curve (CIC) | rmda |

Supplemental Table S3. AUCs of the radiomics scores and prediction models.

|  | Training set | | Internal test set | | External test set | | Prospective test set | |
| --- | --- | --- | --- | --- | --- | --- | --- | --- |
|  | AUC | *p* value | AUC | *p* value | AUC | *p* value | AUC | *p* value |
| Tumor score vs. LN score | 0.80 vs. 0.91 | < 0.01* | 0.76 vs. 0.85 | 0.15 | 0.76 vs. 0.88 | < 0.01* | 0.81 vs. 0.91 | 0.04* |
| Tumor score vs. Clinical model | 0.80 vs. 0.78 | 0.41 | 0.76 vs. 0.79 | 0.74 | 0.76 vs. 0.73 | 0.52 | 0.81 vs. 0.75 | 0.25 |
| Tumor score vs. Clinical-radiomics model | 0.80 vs. 0.94 | < 0.01* | 0.76 vs. 0.92 | < 0.01* | 0.76 vs. 0.92 | < 0.01* | 0.81 vs. 0.95 | < 0.01* |
| LN score vs. Clinical model | 0.91 vs. 0.78 | < 0.01* | 0.85 vs. 0.79 | 0.20 | 0.88 vs. 0.73 | < 0.01* | 0.91 vs. 0.75 | < 0.01* |
| LN score vs. Clinical-radiomics model | 0.91 vs. 0.94 | < 0.01* | 0.85 vs. 0.92 | 0.02* | 0.88 vs. 0.92 | < 0.01* | 0.81 vs. 0.95 | 0.04* |
| Clinical model vs. Clinical-radiomics model | 0.78 vs. 0.94 | < 0.01* | 0.79 vs. 0.92 | 0.01* | 0.73 vs. 0.92 | < 0.001* | 0.75 vs. 0.95 | < 0.01* |

AUC, area under the receiver operating characteristic curve; LN, lymph node. **p* < 0.05.

Supplemental Table S4. Evaluation of clinic-radiomics model and clinical model through NRI and IDI.

| Characteristic | Clinic-radiomics model vs. Clinical model | *p* value |
| --- | --- | --- |
| Training Set |  |  |
| Categorical NRI (95% CI) | 0.272 (0.164, 0.380) | < 0.01* |
| Continuous NRI (95% CI) | 1.344 (1.194, 1.493) | < 0.01* |
| IDI (95% CI) | 0.350 (0.296, 0.403) | < 0.01* |
| Internal Test Set |  |  |
| Categorical NRI (95% CI) | 0.404 (0.138, 0.669) | < 0.01* |
| Continuous NRI (95% CI) | 1.279 (0.956, 1.603) | < 0.01* |
| IDI (95% CI) | 0.385 (0.263, 0.507) | < 0.01* |
| External Test Set |  |  |
| Categorical NRI (95% CI) | 0.246 (0.112, 0.379) | < 0.01* |
| Continuous NRI (95% CI) | 1.339 (1.144, 1.535) | < 0.01* |
| IDI (95% CI) | 0.353 (0.287, 0.419) | < 0.01* |
| Prospective Test Set |  | < 0.01* |
| Categorical NRI (95% CI) | 0.693 (0.489, 0.900) | < 0.01* |
| Continuous NRI (95% CI) | 1.624 (1.412, 1.836) | < 0.01* |
| IDI (95% CI) | 0.462 (0.367, 0.556) | < 0.01* |

CI, confidence interval; IDI, integrated discrimination improvement; NRI, net reclassification improvement.**p* < 0.05.

Supplemental Table S5. The performance of radiologists without and with AI assistance in the prospective test set.

|  | AUC  (95% CI) | ACC | SEN | SPE | PPV | NPV |
| --- | --- | --- | --- | --- | --- | --- |
| Radiologists without AI assistance |  |  |  |  |  |  |
| Junior | 0.71  (0.62-0.79) | 70.1% | 79.6% | 61.9% | 64.2% | 78.0% |
| Senior | 0.75  (0.66-0.83) | 76.1% | 63.0% | 87.3% | 81.0% | 73.3% |
| Radiologists with AI assistance |  |  |  |  |  |  |
| Junior | 0.82  (0.74-0.88) | 82.1% | 77.8% | 85.7% | 82.4% | 81.8% |
| Senior | 0.85  (0.78-0.91) | 86.3% | 72.2% | 98.4% | 97.5% | 80.5% |

Acc, accuracy; AI, artificial intelligence; AUC, area under the receiver operating characteristic curve; NPV, negative predictive value; PPV, positive predictive value; SEN, sensitivity; SPE, specificity.
